# Monitoring Inhibition of Hemoglobin Peroxidase Activity After Exposure to Cigarette Smoke Using an Electrochemical Biosensor

**DOI:** 10.3390/bios15120767

**Published:** 2025-11-25

**Authors:** Alfonso Sequeda-Juárez, Flor Cortés-Ortegón, Diego Ortega-Picazo, José Antonio García-García, Ana María Espinosa-García, Celia Sánchez-Pérez

**Affiliations:** 1 Laboratorio de Dispositivos Biomédicos, Instituto de Ciencias Aplicadas y Tecnología, Universidad Nacional Autónoma de México, Circuito Exterior s/n, Ciudad Universitaria, Mexico City 04510, Mexico; alfonso.sequeda@icat.unam.mx (A.S.-J.); flor.cortes@icat.unam.mx (F.C.-O.); diego.ortega@icat.unam.mx (D.O.-P.); 2 Dirección de Enseñanza, Hospital General de México “Dr. Eduardo Liceaga”, Mexico City 06726, Mexico; drjagarcia2@prodigy.net.mx; 3 Laboratorio de Biología Molecular, Dirección de Investigación, Hospital General de México “Dr. Eduardo Liceaga”, Mexico City 06726, Mexico; anaesga@hotmail.com

**Keywords:** hemoglobin, peroxidase activity, biosensor, electrochemical impedance spectroscopy, cyclic voltammetry, chronoamperometry, pollutants, cigarette smoke

## Abstract

This work presents a catalysis-based electrochemical biosensor to evaluate the peroxidase-like activity of methemoglobin (Hb-PLA) after exposure to cigarette smoke (CS) at different time intervals. The system consists of a microelectrode array coupled with a PDMS chamber containing a methemoglobin solution (biorecognition element). Hydrogen peroxide (H_2_O_2_) acts as the substrate, while 3,3′,5,5′-tetramethylbenzidine (TMB) functions as the chromogenic substrate for the Hb-PLA through its oxidation reaction. A spectrophotometric technique is used as a reference method to assess the catalytic activity of methemoglobin. Positive control samples exhibited higher absorbance, indicating strong catalytic activity, whereas CS-exposed samples showed a marked reduction, which was confirmed by the negative control. Cyclic voltammetry revealed significant alterations in the oxidation and reduction peaks of the CS-exposed samples. Therefore, chronoamperometry was employed to quantify the charge transfer as the electrochemical response associated with Hb-PLA, yielding a sensitivity of 0.86 ± 0.06 (%Hb-PLA/mC) and a limit of detection (LOD) of 0.23 (mC). The results demonstrate that cigarette smoke impairs the Hb-PLA in a time-dependent manner, with longer exposure reducing the activity by up to 25%. The proposed biosensor provides a rapid, sensitive, and straightforward strategy for detecting functional alterations in solutions of methemoglobin induced by environmental pollutants such as cigarette smoke.

## 1. Introduction

Hemoglobin (Hb) is a protein found within erythrocytes, and it is responsible for transporting oxygen from lungs to tissues via the bloodstream [1]. Hb is a multifunctional molecule involved in various processes, including catalytic functions (such as nitrite reductase, dioxygenase, monooxygenase, alkyl hydroperoxidase, esterase, and lipoxygenase activities), nitric oxide metabolism, metabolic reprogramming, pH regulation, and redox homeostasis [2]. Due to its diverse roles, Hb is of great interest in the medical field, as it may contribute to the understanding of physiological processes associated with diseases. Its catalytic activity has been widely studied and has proven useful in the diagnosis of diseases linked to environmental pollutants, such as cigarette smoke exposure [3,4].

Cigarette smoking is associated with an increased risk of developing pulmonary and cardiovascular diseases, obesity, and various types of cancer, including liver and pancreatic cancer. Notably, approximately 90% of lung cancer cases are directly linked to tobacco use [5]. Among the mechanisms triggered by cigarette smoke (CS) is oxidative stress, which involves the generation of reactive oxygen species (ROS), free radicals, and other oxidizing agents resulting from an imbalance between pro-oxidant and antioxidant systems [6].

The main ROS generated by cigarette smoke are products of lipid peroxidation, such as 4-hydroxynonenal (4-HNE), malondialdehyde (MDA), and 8-isoprostane. Additionally, oxidatively modified protein by-products, including protein carbonyls, 3-nitrotyrosine, and oxidized α-1 antitrypsin, as well as oxidized nucleic acid metabolites like 8-hydroxy-2′-deoxyguanosine (8-OHdG), are also involved. Changes in antioxidant levels, such as reduced glutathione (GSH), are also observed [7].

Hemoglobin can eliminate several of these species derived from cigarette smoke through its peroxidase-like activity (Hb-PLA). In its methemoglobin form, it acts as a redox mediator in reactions with hydrogen peroxide (H_2_O_2_) forming ferryl hemoglobin (Fe^4+^ = O) that promote oxidation of other molecules. This process contributes to both the detoxification of reactive oxygen species and, under certain conditions, to autoxidation and oxidative damage, as represented by the following reaction::

Hb (Fe^3+^) + H_2_O_2_→Hb(Fe^4+^) + H_2_O (1)

The peroxidase-like activity of methemoglobin (Hb) plays a protective role in cells by decomposing hydrogen peroxide into less reactive species, thereby preventing the formation of hydroxyl, hydroperoxyl, phenoxyl, nitrogen dioxide, and lipid radicals. These reactive species contribute to oxidative stress and damage to macromolecules, ultimately leading to cell death [7].

Under conditions of elevated hydrogen peroxide (H_2_O_2_) concentrations, methemoglobin (Hb) undergoes autoxidation, which destabilizes the tertiary structure of the globin chains and can lead to the formation of reactive intermediates such as the ferryl/ferryl protein radical species (·HbFe^4+^ = O). This oxidative species can damage amino acid residues within the protein, including the irreversible oxidation of βCys93. Such modifications have a destabilizing effect that contributes to Hb unfolding, dissociation into dimers, increased rates of autoxidation, and rapid heme loss—processes with significant implications for health [7,8].

Various devices have been developed to measure free radicals and other harmful substances derived from cigarette smoke, based on their optical, electrical or mass properties [9]. Biosensors have been widely used in the healthcare field for diagnosis, prognosis, monitoring and evaluating treatment responses in a variety of diseases. These devices enable real-time characterization of biological samples with high precision and reliability, offering sensitive and specific detection capabilities. The peroxidase-like activity of methemoglobin (Hb-PLA) has been analyzed using biosensors to assess its electrochemical and electrocatalytic behavior, correlating it with Hb-PLA activity through the reaction with H_2_O_2_. Techniques such as cyclic voltammetry (CV), amperometry, and electrochemical impedance spectroscopy (EIS) have been employed for this purpose [10,11]. Recently, electrochemical biosensors have been proposed to detect nicotine and other cigarette-derived compounds [12,13]. However, to date, the effect of cigarette smoke exposure on the Hb-PLA has not been investigated nor quantified.

In this study, we propose a catalysis-based biosensor designed to measure the peroxidase-like catalytic activity of methemoglobin (Hb-PLA) mediated by the redox reaction between H_2_O_2_ and TMB. The resulting catalytic process is electrochemically transduced through a microelectrode array, enabling the biorecognition of this specific catalytic activity of Hb. An absorbance spectrophotometric technique was first used as reference to validate Hb peroxidase like activity by monitoring the oxidation of 3,3′,5,5′-tetramethylbenzidine (TMB) by the hydrogen peroxide (H_2_O_2_) at 660 nm. Subsequently, we use cyclic voltammetry (CV) to qualitatively analyze and validate the redox behavior associated with H_2_O_2_ catalysis. Finally, chronoamperometry (CA) was employed to quantify charge transfer, allowing comparison among the positive control, cigarette smoke CS-exposed, and negative control samples. This approach enabled the correlation of catalytic activity loss with the total transferred charge, providing a quantitative electrochemical readout of the Hb-PLA response upon exposure to cigarette smoke. This detection method could help assess toxicity levels in the body caused by increased reactive oxygen species (ROS), well known as contributors to cellular and tissue damage [14,15]. This sensing approach may lead to a fast device for measuring Hb-PLA in smokers, offering a valuable tool for the investigation and monitoring of smoke-related diseases.

## 2. Materials and Methods

This study was conducted using methemoglobin, as its ferric form (Fe^3+^) allows it to behave chemically in a manner similar to peroxidase enzymes, enabling it to catalyze peroxides by reacting with H_2_O_2_ to form an oxoferryl species (Fe^4+^). In this way, the catalyzed H_2_O_2_ can oxidize organic molecules such as TMB. Thus, the peroxidase-like activity of methemoglobin (Hb-PLA) was assessed through spectrophotometric and electrochemical methods. As a reference method for the Hb-PLA measurement, TMB oxidation by H_2_O_2_ in the presence of methemoglobin was monitored by UV–Vis spectroscopy, while cyclic voltammetry and chronoamperometry were applied to evaluate the redox behavior and quantify charge transfer associated with Hb catalytic activity. A positive control consisting of methemoglobin and TMB in the presence of H_2_O_2_ was tested, whereas a negative control was prepared by adding sodium azide (NaN_3_), an inhibitor of methemoglobin’s catalytic activity. Both positive and negative controls were maintained under ambient conditions, without exposure to cigarette smoke. The experimental design was optimized to evaluate the time-dependent effect of cigarette smoke on Hb-PLA activity. Accordingly, exposure intervals were strictly controlled, and the internal smoke pressure within the chamber was kept constant. This study does not attempt to quantify smoke concentration, rather the set up was designed to ensure reproducible exposure conditions in order to compare the catalytic activity across different exposure times.

### 2.1. Hemoglobin Sample Preparation

**Figure 1:**
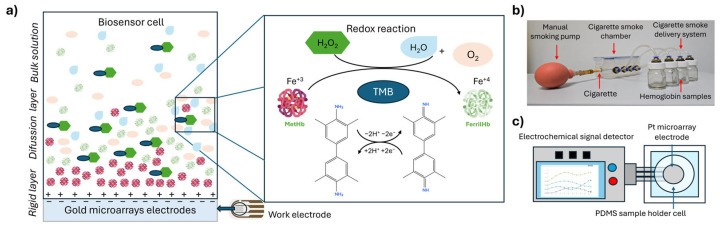
Description of the electrochemical sensor for (**a**) Diagram illustrating the peroxidase like activity of the methemoglobin, (**b**) System for exposing methemoglobin solutions to cigarette smoke where the differential pressure inside the chamber was maintained a 7 ± 1 Pa. and (**c**) Schematic representation of the biosensor: microelectrode array within an electrochemical cell containing the solutions to be measured.

Methemoglobin solutions (**Hb**) were prepared by mixing lyophilized methemoglobin (Sigma-Aldrich, Saint Louis, MO, USA) in phosphate-buffered saline (PBS, pH 7.4) to a final concentration of 250 µg/mL (3.88 μM). An aliquot of this solution was used as the positive control (**Hbs**). A negative control (**Hb_AZ_**) was prepared from another aliquot by adding sodium azide (NaN_3_, Karal, Guanajuato, Mexico) in a concentration of 15 nM. The **Hb_AZ_** sample was incubated for 10 min at room temperature. Similarly, three aliquots of the **Hb** solution were directly exposed to commercial cigarette (Link, Mexico City, Mexico) smoke (**Hb_CS_**) using the experimental setup shown in Figure 1b, with exposure times (t_CS_) of 1, 5, and 10 min. These samples were labeled **Hb_CS1_**, **Hb_CS5_**, and **Hb_CS10_**, respectively. For the optical and electrochemical measurements of Hb-PLA activity, hydrogen peroxide (H_2_O_2_) and the chromogenic substrate 3,3′,5,5′-tetramethylbenzidine (TMB, Life Technologies, Waltham, MA, USA) were added to each sample at a 1:10 ratio. This reaction catalyzes the H_2_O_2_ oxidizing of TMB. The resulting samples with H_2_O_2_ and TMB additionally added were labeled as: *Hb_S_ y *Hb_AZ_ for the positive and negative controls, respectively, and *Hb_CS1_, *Hb_CS5_, *Hb_CS10_ for the smoke-exposed samples.

### 2.2. Measurement of Hb Catalytic Activity by UV–Vis Spectrophotometry

To study the peroxidase-like activity of methemoglobin (Hb-PLA), the oxidation of the TMB substrate was monitored in the presence of 5 µL of 10 mM H_2_O_2_ in the following solutions: *Hb_S_, cigarette smoke-exposed solutions (*Hb_CS_), and the negative control (*Hb_AZ_). This was carried out by acquiring absorbance spectra using an experimental setup consisting of a DH-2000 halogen light source, a 1 cm^2^ cuvette holder (using 1mL volume sample), and a Flame UV-VIS-NIR spectrophotometer both from Ocean Optics, Orlando, FL, USA. Subsequently, relative spectra ($A_{r,i}$) were calculated by normalizing the absorbance spectra (Ai) of the *Hb_S_, *Hb_CS_, and *Hb_AZ_ samples with respect to the spectrum of the methemoglobin reference sample (AHbs).

(2) $$A_{r,i}=\frac{Ai}{AHbs},$$

where Ai corresponds to the absorbance spectra of *Hb_S_, *Hb_CS_, and *Hb_AZ_.

From the relative absorbance spectra, the values at the characteristic maximum absorption wavelength of TMB (λmax = 660 nm) were extracted. The characteristic time of the Hb-PLA kinetic activity (t_Km_) for each sample was calculated using the Michaelis–Menten equation, considering a total monitoring time (t_m_) of 30 min, which allowed the determination of the corresponding Michaelis-Menten constant (K_m_). Finally, the percentage of catalysis for the *Hb_CS1_, *Hb_CS5_, *Hb_CS10_, and *Hb_AZ_ samples were calculated relative to the catalysis observed in the *Hb_S_ sample, which served as the positive control.

### 2.3. Characterization of the Catalytic Activity of Hb Using the Electrochemical Biosensor

The G-IDE555 platinum interdigitated microelectrode (Metroohm Dropsens, S.L.U., Asturias, Spain) was used for the electrochemical measurement of methemoglobin catalytic activity. Prior to use, the electrode was pretreated with 0.5 M H_2_SO_4_ by applying a potential sweep from −0.5 V to 1.0 V to clean the electrode surface. The electrode was then placed on a glass slide and sealed with a custom-made polydimethylsiloxane (PDMS) (Dow Chemical Company, Sylgard^TM^ 184 Silicone Elastomer Kit, Hergestellt, Midland, MI, USA) cell that served as the sample chamber. In this latter, 45 µL of the Hb_S_, Hb_CS_, and Hb_AZ_ solutions were placed, serving as bioreceptors in the electrochemical system. Hydrogen peroxide (H_2_O_2_) and TMB were subsequently added as analytes to evaluate the Hb-PLA catalytic activity (Figure 1c). Electrochemical characterization was performed using the PalmSens4 potentiostat and data analysed with PSTrace 5.11.1006 (PalmSens, Houten, The Netherlands).

The electrical response of Hb-PLA was characterized via cyclic voltammetry (CV) using 45 µL of the *Hb_S_, *Hb_CS_, and *Hb_AZ_ solutions at different monitoring times (t_m_) to observe their electrochemical evolution. Measurements were performed under optimized conditions with the applied current controlled within a range of 100 nA to 1 mA, an equilibrium time of 5 s, and a potential window from −1.0 V to 1.0 V, with a scan rate of 0.01 V/s. Chronoamperometry (CA) was also used to quantify the Hb-PLA activity in *Hb_S_ and *Hb_CS10_ samples, as well as in the negative control (*Hb_AZ_). For these measurements, we used optimized parameters: a current range of 0.1 to 1.0 mA was used with a signal of 0.5 V_DC_, an equilibrium time of 4 s, a measurement interval of 5 s, and a total duration of 1200 s (20 min). A 40 µL aliquot of the *Hb_CS5_ sample was added to the PDMS chamber, and after the equilibrium time was reached, 5 µL of 10 mM H_2_O_2_ was added to initiate the peroxidase activity reaction. Finally, we calculated the current as a function of time and determined the accumulated charge Q over time using the following equation:

(3) $$Q=\int_{t_{0}}^{t_{k}}I\left(t\right)\;dt,$$

where *I*(*t*) is the measured current and *t_0_* is the initial time and *t_k_* is the time elapsed until measurement.

## 3. Results

### 3.1. Optical Characterization of Hb-PLA Activity After Cigarette Smoke Exposure

**Figure 2:**
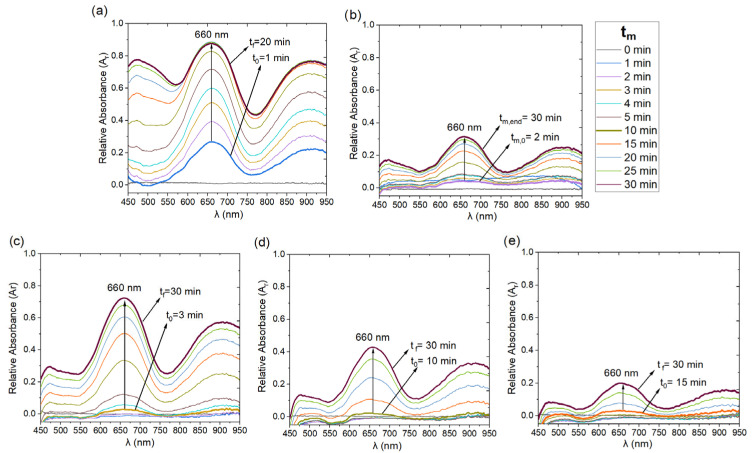
Relative absorbance (Ar) spectra at different measurement times (tm) for: (**a**) positive control *Hbs (Hb + TMB + H_2_O_2_ under ambient conditions), (**b**) negative control *Hb_AZ_ (Hb + NaN_3_ + TMB + H_2_O_2_ under ambient conditions) and methemoglobin solutions (Hb + TMB + H_2_O_2_) exposed to cigarette smoke (CS) for times of (**c**) 1 min (*Hb_CS1_), (**d**) 5 min (*Hb_CS5_), and (**e**) 10 min (*Hb_CS10_).

Figure 2 shows the relative absorbance spectra ($A_{r}$) of the *Hb_S_, *Hb_CS_, and negative control *Hb_AZ_ samples, measured over a 30-min period. Spectra were recorded every minute during the first 5 min and every 5 min thereafter. The spectrum $A_{r*Hb\;}$ in Figure 2a displays the characteristic spectral profile of TMB oxidation in the presence of H_2_O_2_, with the reaction initiating at t_m,0_ = 1 min and an absorbance value $A_{r660}$= 0.26. The signal increased progressively over time, reaching a maximum $A_{r660}$ = 0.88 at 30 min. This result reflects the catalytic activity of MetHb in an oxidative environment promoted by H_2_O_2_, a strong inducer of reactive oxygen species (ROS). In contrast, the relative absorbance spectrum of the negative control *Hb_AZ_ (Figure 2b) showed no significant change until t_m,0_ = 2 min, at which point a similar spectral profile to that of *Hb_S_ was observed. However, the $A_{r660}$ values were notably lower, indicating slower catalysis, reaching a maximum of only $A_{r660}$ = 0.31 at t_m,end_ = 30 min. This behavior suggests inhibition of Hb-PLA activity due to the presence of sodium azide (NaN_3_).

To investigate the effect of cigarette smoke exposure on methemoglobin, the relative absorbance spectra of *Hb_CS_ samples were recorded following the exposure procedure described in Section 2.1. Figure 2c–e show the $A_{r}$ spectra for *Hb_CS1_, *Hb_CS5_, and *Hb_CS10_, respectively. Spectral monitoring of Hb-PLA activity in these samples revealed a delayed onset of catalytic activity, with t_m,0_ values of 3, 10, and 15 min, respectively. After 30 min of monitoring, reduced activity was observed in all *Hb_CS_ samples, with $A_{r660}$ values of 0.72, 0.42, and 0.20 for *Hb_CS1_, *Hb_CS5_, and *Hb_CS10_, respectively. Compared to the *Hb_S_ control (Figure 2a), these findings suggest cigarette smoke exposure inhibits Hb-PLA activity in a time-dependent manner.

**Figure 3:**
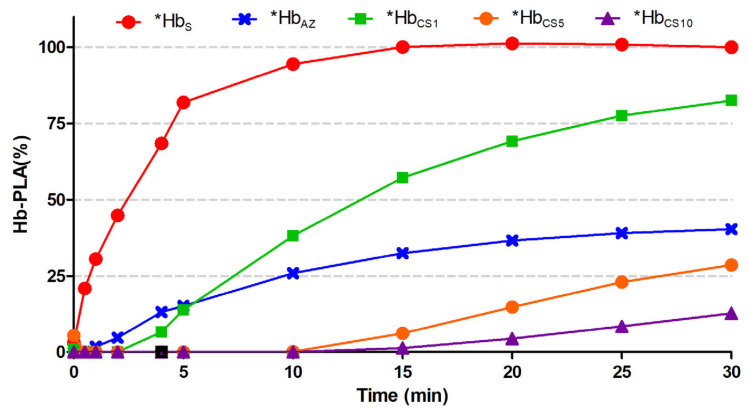
Percentage change in Hb-PLA activity and its uncertainty as a function of the measurement time (tₘ) from 0 to 30 min, taken from the relative absorbance spectra for the positive control *Hbs (Hb + TMB + H_2_O_2_), negative control *Hb_AZ_ (Hb + NaN_3_ + TMB + H_2_O_2_) and methemoglobin solutions (Hb + TMB + H_2_O_2_) exposed to cigarette smoke for times of 1 min (*Hb_CS1_), 5 min (*Hb_CS5_), and 10 min (*Hb_CS10_).

To quantify this inhibitory effect over short-term exposure durations (t_m_ = 0–30 min), the percentage change in peroxidase-like activity (%Hb-PLA) was calculated relative to the activity of the *Hb_S_ sample. As shown in Figure 3, the positive control (*Hb_S_) displayed a rapid increase in activity, reaching its maximum at 15 min. In contrast, the *Hb_CS_ samples exhibited extended periods of inactivity, with %Hb-PLA = 0 between t_m,0_ = 1 and 15 min for *Hb_CS1_ and *Hb_CS10_, respectively. After these delays, peroxidase activity gradually resumed but with slower kinetics as cigarette smoke exposure time increased. Notably, none of the *Hb_CS_ samples recovered full Hb-PLA activity within the 30-min monitoring period, as their maximum %Hb-PLA value remained well below that of the positive control.

### 3.2. Kinetic Analysis of Hb-PLA Activity

Table 1 presents the maximum %Hb-PLA values and the characteristic kinetic times (t_Km_) for each sample. The *Hb_S_ sample reached 100% activity at 30 min and achieved 50% of this activity at t_Km_ = 2 min. In contrast, the negative control *Hb_AZ_ showed a maximum %Hb-PLA of 43%, with a t_Km_ = 17.1 min. Among the smoke-exposed samples, *Hb_CS1_ achieved a maximum %Hb-PLA of 72% with a t_Km_ = 10.1 min, lower than *Hb_S_ but higher than *Hb_AZ_. In the cases of *Hb_CS5_ and *Hb_CS10_, the maximum %Hb-PLA was below 43% and 20%, respectively. Neither sample reached 50% activity within the monitored timeframe, preventing calculation of a defined t_Km_. These results indicate that *Hb_CS10_ exhibited the most pronounced inhibition of Hb-PLA activity under the tested conditions. Overall, the data suggest that cigarette smoke exposure leads to inhibition of Hb-PLA activity, which becomes more severe with longer exposure durations. Furthermore, none of the smoke-exposed samples were able to recover the full catalytic activity observed in the *Hb_S_ control, suggesting a quasi-reversible inhibitory effect of the peroxidase like catalytic activity under the conditions studied.

### 3.3. Measurement of Hb-PLA Activity by Cyclic Voltammetry

**Figure 4:**
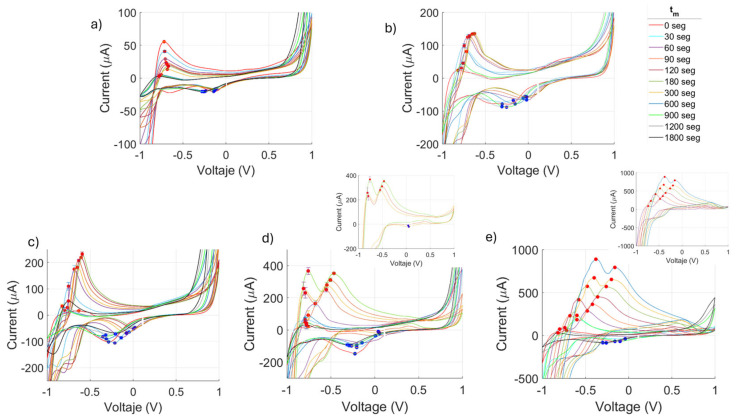
Kinetics of methemoglobin peroxidase activity measured by cyclic voltammetry (CV). Voltamograms for the (**a**) positive control *Hbs (Hb + TMB + H_2_O_2_) and (**b**) the negative control *Hb_AZ_ (Hb + NaN_3_ + TMB + H_2_O_2_) from 30 s to 1800 s. Smoke-exposed solutions (Hb + TMB + H_2_O_2_) are shown for (**c**) 1 min *Hb_CS1_, (**d**) 5 min *Hb_CS5_, and (**e**) 10 min *Hb_CS10_, from 60 to 600 s.

The results obtained through cyclic voltammetry (CV) using the biosensor with a microelectrode array are shown in Figure 4 and data included in Appendix A. These results display redox behavior in both the positive and negative controls, as well as in the *Hb_CS1_ sample with the shortest exposure time to CS. Figure 4a presents the voltammogram of the *Hb_S_ sample, which exhibited a stable response with a decrease in the oxidation peak current (I_po_) from 54.0 to 1.6 μA at approximately the same peak potential (V_po_) of −0.8 V. A decrease in the reduction peak current (I_pr_) was also observed, from −19.1 to −16.6 μA for a first period of the monitoring time (t_m_) from 30 to 180 s, then stabilized at I_pr_= −19.9 μA from t_m_= 300 to 1800 s at V_pr_ = −0.3 V.

The negative control *Hb_Az_ (Figure 4b) exhibited moderately higher oxidation and reduction activity over time compared to *Hb_S_. During t_m_ = 30–300 s, the oxidation peak current (I_po_) increased from 98.8 to 135.5 μA at potentials between V_po_ −0.8 and −0.6 V. In the later period (t_m_ = 600–1800 s), I_po_ decreased from 81.2 to 30.7 μA, while the reduction peak current (I_pr_) ranged from −78.3 to −85.9 μA at V_pr_ ~−0.3 V.

The *Hb_CS1_ sample exhibited pronounced redox processes, with oxidation peak currents (I_po_) increasing from 16 to 232.3 μA during the first 180 s at potentials of V_po_ = −0.6 to −0.5 V. In the same period, reduction peak currents (I_pr_) ranged from –101.1 to –47.1 μA within a similar potential window (V_pr_ = −0.28 to −0.02 V). During the second interval (300–1800 s), I_po_ decreased from 181.1 to 53.9 μA (V_po_ = −0.6 to −0.7 V), while I_pr_ varied from −68.7 to −71.8 μA (V_pr_ = −0.7 to −0.2 V). It is worth to note that the reduction behavior of this sample resembled that of the negative control.

However, samples *Hb_CS5_ and *Hb_CS10_, with longer CS exposure times, showed altered redox behavior, with more pronounced changes during the course of the peroxidase-like catalytic reaction, especially with increasing exposure duration. Notably, *Hb_CS5_ (Figure 4c) exhibited a transformation of the oxidation peak into a double oxidation process, which appeared from t_m_ = 120 s and persisted until 300 s, as shown in the inset graph. In *Hb_CS10_ (Figure 4d), this double oxidation process was even more pronounced, starting at 60 s and lasting until 600 s, with a longer duration than in the previous case (see the inset).

For both samples, these double oxidation processes were accompanied by a loss of the reduction process, which was later restored, returning to a single redox process similar to the initial state. For *Hb_CS5_ (Figure 4d), at the beginning of monitoring (t_m_ = 30–90 s), the redox activity showed I_po1_ values ranging from 64.5 to 251.1 μA (V_po1_ = −0.79 to −0.55 V) and I_pr1_ = −108.4 to −36.7 μA (V_pr1_ = −0.2 to −0.01 V). During the double oxidation phase (t_m_ = 120 to 300 s, see the inset), the first oxidation peak reached I_po1_ = 230.5 to 366.9 μA, and the second peak reached I_po2_ = 310.4 to 351.9 μA, with no observable reduction process. In the final phase of monitoring (t_m_ =600–1800 s), there is a return to a decreased redox activity with I_po_ = 91.84 to 25.89 μA at a nearly V_po_ = 0.75 V. The current reduction showed minimal variation in the last period, with I_pr_ ~−95 μA at V_pr_ ~−0.25 V.

Finally, the *Hb_CS10_ sample (Figure 4e) initially t_m_ = 30 s exhibited redox activity with I_po_ = 184 μA and I_pr_ = −39 μA at V_po_ = −0.5 V and V_pr_ = −0.04 V. In the same way as *Hb_CS5,_ this sample exhibited a double oxidation process. In this double peak phase (t_m_ = 60–600 s), the first peak reached I_po1_ = 90.4 to 888.2 μA, and the second **I_po2_** = 285.92 to 793 μA. This double oxidation required higher potential: **V_po1_** = −0.4 to −0.2 V and **V_po2_** = −0.7 to −0.4 V for the first and second peaks, respectively. In the final phase of monitoring, there is also a return to the redox activity that reached **I_po_** = 233.8 to 30.5 μA at **V_p_** = −0.6 to −0.81 V. The corresponding reduction occurred with **I_pr_** = −68.2 to −84.4 μA at **V_pr_** = −0.9 to −0.2 V.

These results suggest that CS exposure enhances the oxidative activity of Hb, as evidenced by the progressive loss of its reductive capacity. On the other hand, in the *Hb_CS1_ sample, the reduction-related values were initially more negative than in the negative control *Hb_AZ_ (up to −105.72 μA), but gradually stabilized over time. In *Hb_CS5_ and *Hb_CS10_, CS exposure altered the redox process from a single to a double oxidation event, with increased persistence over time as exposure increased. This was accompanied by a loss of the reduction capacity.

The redox process occurs primarily in methemoglobin, while TMB acts as the chromogenic substrate in this reaction. The oxidizing intermediates produced during catalysis transfer electrons to TMB, oxidizing it and producing the characteristic blue color change.

In the cyclic voltammograms, the anodic peaks correspond to the oxidation of methemoglobin (oxoferryl form), forming oxidized species in the presence of hydrogen peroxide known as ferryl Hb. The cathodic peaks, in turn, reflect the reduction in these previously oxidized ferryl Hb species back to the methemoglobin form, thus completing a normal catalytic cycle in the absence of inhibitors.

**Figure 5:**
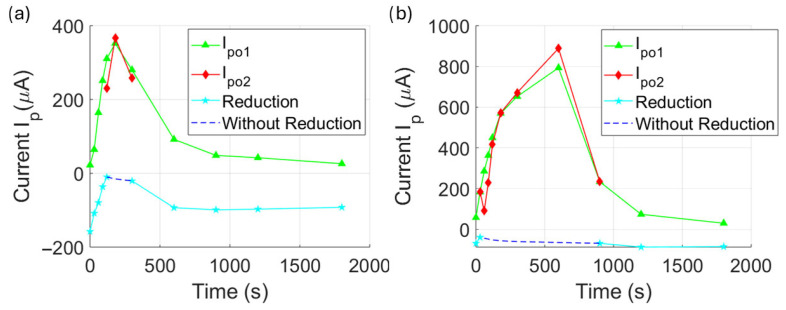
Graphs showing the maximum double oxidation and reduction peaks of hemoglobin peroxidase activity across the measurement time for cigarette smoke exposed solutions (Hb + TMB + H_2_O_2_) for (**a**) 5 min *Hb_C5_. and (**b**) *Hb_C10_.

Upon exposure to CS, inhibition is observed as a decrease in the cathodic peak currents, suggesting an alteration of the peroxidase-like catalytic activity of methemoglobin and an increased formation of oxidized species. Additionally, the emergence of a new cathodic peak indicates the possible formation of new oxidized species due to secondary products derived from the interaction of Hb with toxic agents present in CS possibly related to structural modifications of the protein or the heme group. Figure 5 shows the oxidation peak current values (i.e., positive current measurements), as well as the negative peak values corresponding to the reduction processes. In the *Hb_CS1_ sample, changes in PLA activity were observed, with an increase in oxidation peak during the first two minutes of catalysis, followed by a decrease up to 10 min. After that, a linear increase in current was seen, reaching a maximum of 580 μA at 20 min after the reaction began.

In *Hb_CS5_, the oxidation peak increased at 2 min into the reaction, then decreased and remained stable until 15 min, after which it reached a new peak of approximately 670 μA at 20 min, exceeding the previous exposure time. Finally, *Hb_CS10_ showed a rapid increase in oxidation peaks within the first two minutes of the PLA reaction, with current rising above the values of earlier time points, reaching around 610 μA at 20 min.

In contrast, the reduction peak currents of Hb-PLA activity decreased progressively with longer CS exposure times (5 and 10 min). The increase in oxidation current of CS-exposed Hb, along with the loss of its reduction current, indicates a quasi-reversible process, reflecting a decreased ability of Hb to return to its reduced state.

### 3.4. Measurement of Hb Activity by Chronoamperometry

**Figure 6:**
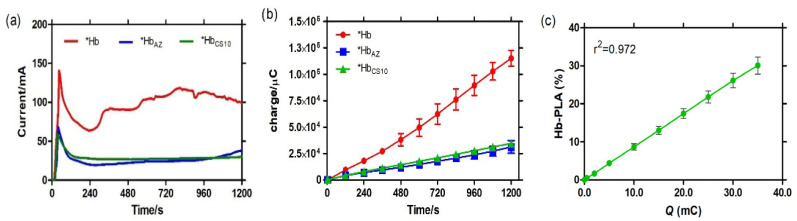
Methemoglobin peroxidase activity (Hb-PLA) by chronoamperometry for positive control *Hbs (Hb + TMB + H_2_O_2_), negative control *Hb_AZ_ (Hb + NaN_3_ + TMB + H_2_O_2_) and 10 min cigarette smoke exposed (Hb + TMB + H_2_O_2_) solutions for (**a**) amperometric curves of Hb-PLA upon addition of 10 mM H_2_O_2_ and (**b**) electric charge during Hb-PLA measurements. (**c**) Percentage of Hb-PLA exposed to CS for 10 min based on electric charge measurements taken over 1200 s.

Chronoamperometric (CA) results show the time-dependent current response of the Hb-PLA system. Under control conditions, an increase in current up to 140 µA was observed, followed by slow oscillations after approximately 240 s from the start of the reaction. In contrast, the Hb-PLA systems exposed to sodium azide (inhibitor) and cigarette smoke (CS) exhibited similar behaviors, with maximum current peaks of 60 µA and 70 µA, respectively, during the first 30 s of the measurement. After 240 s, both conditions stabilized below 30 µA. At the end of the experiment (1200 s), the control condition showed a slight increase in current, reaching 36 µA (Figure 6a). Figure 6b shows the accumulated charge (Q) as a function of time. Under control conditions, the Hb-PLA exhibited a linear increase, reaching an average charge of 115 mC at 1200 s. In contrast, the Hb-PLA when samples were exposed to sodium azide (*H_AZ_) and cigarette smoke showed charge values below 32 mC throughout the entire measurement period.

Based on the results obtained by spectrophotometry, the percentage of Hb-PLA in the biosensor was determined using the accumulated charge values. The maximum percentage of Hb-PLA was calculated at 1200 s, a time at which the increase in activity remained linear. The plot showed an increase in Hb-PLA up to 90% at 600 s, while the Hb exposed to CS increased only up to 33% (Figure 6c). The results obtained by chronoamperometry were consistent with those measured by UV–Vis spectroscopy, where the activity remained below 25% at 1200 s. Table 2 shows the comparison between the electrochemical (biosensor-based) and optical methods used to evaluate the Hb-PLA activity after exposure to cigarette smoke.

## 4. Discussion

Hemoglobin plays a critical role in physiological processes, functioning not only as the primary oxygen transporter from the bloodstream to peripheral tissues but also as a peroxidase capable of reducing substrates such as H_2_O_2_ [16]. This activity helps prevent the generation of free radicals that can damage hemoglobin itself as well as lipids, proteins, and DNA, ultimately contributing to cellular dysfunction and tissue injury, particularly in cardiac and skeletal muscle. While hemoglobin shares peroxidase-like activity with enzymes such as cytochrome P450, catalase, prostaglandin H synthase, and photosystem II—where radical formation is tightly regulated within their catalytic cycles—hemoglobin is unable to efficiently control radical generation, resulting in unavoidable leakage and harmful oxidative reactions [17]. The biosensor developed in this study allowed in situ monitoring of Hb catalytic activity under exposure to cigarette smoke (CS), revealing marked alterations in its redox behavior. These changes were evident in both spectrophotometric analyses of Hb-PLA activity and in voltametric and chronoamperometric profiles obtained using the biosensor.

### 4.1. Spectrophotometry

Previous studies have demonstrated the peroxidase activity of Hb using colorimetric substrates through H_2_O_2_ catalysis but this effect have not been studied under exposure of the Hb to CS. Some other approaches have enhanced Hb peroxidase activity by forming complexes, such as binding with tartaric acid, yielding activities comparable to horseradish peroxidase [18]. In our study, 3,3′,5,5′-tetramethylbenzidine (TMB) was used as the redox substrate. Under normal conditions, Hb displayed efficient peroxidase activity, initiating rapidly upon H_2_O_2_ addition. However, exposure to CS drastically reduced Hb peroxidase activity in a time-dependent manner, with near-complete loss of this activity after 10 min of exposure.

Findings reported in the literature show that the inhibition of methemoglobin (Hb) by cigarette smoke could be attributed to the presence of reactive oxygen species ROS and nitrogen species RNS as well as toxic gases such as carbon monoxide (CO) and nitrogen oxides. The effect of these compounds have been reported as they oxidize the heme iron (Fe^2+^/Fe^3+^) and generate ferryl intermediates (Fe^4+^ = O), causing structural and functional alterations that impair the peroxidase-like redox cycle of methemoglobin [15,19]. Similar inhibitory effects of CS on peroxidase activity have been reported in oral peroxidase, which plays a key role in neutralizing free radicals generated by tobacco use and has been associated with oral cancer development [20]. In this study, the results showed that the duration of Hb exposure to CS was critical, with prolonged times inducing a quasi-reversible Hb-PLA. For instance, 1 min of CS exposure led to a rapid, reversible inhibition of approximately 90% cause the %Hb-PLA reaches 10% during the five minutes of the reaction, whereas more than 5 min of exposure caused a greater loss of %Hb-PLA, with activity reduced to 0% for 10 min and only partial recovery (25% and 12%) was reached at 25 and 30 min, respectively. In a previous study employing different inhibitors, including NaN_3_, H_2_O_2_, and HCl, peroxidase activity was reduced by up to 80% after 20 min of exposure. Furthermore, inhibition by NaN_3_ and H_2_O_2_ was described as partially reversible, whereas HCl induced irreversible inhibition [21].

These findings are consistent with reports showing that CS induces oxidative stress and the release of thiocyanates, compounds linked to peroxidase inhibition. These alterations indicate profound physicochemical changes in Hb among smokers. Notably, the average time required to smoke a single cigarette (5–7 min) aligns with the exposure times in which we observed quasi-reversible inhibition of Hb-PLA [22]. Prolonged exposure is also known to increase carboxyhemoglobin saturation (SpCO) by up to 5%, which remains elevated for extended periods after smoking, further supporting our observations of inhibition [23]. Under these conditions, CO binding stabilizes Hb in its ferrous form (Fe^2+^), preventing peroxidase activity that requires ferric iron (Fe^3+^), while simultaneously contributing to systemic oxidative stress by inhibiting other peroxidases [24].

Several studies have also reported hematological alterations in smokers, including increased white blood cell counts, elevated Hb concentration, hematocrit, and mean corpuscular hemoglobin. These hematological changes correlate with exposure duration, underscoring the relevance of assessing Hb catalytic activity in relation to smoking [25]. The growing development of biosensors for biological monitoring further highlights their utility, not only for disease diagnostics (e.g., cancer and diabetes) but also for evaluating health impacts of environmental exposures such as CS [26].

### 4.2. Cyclic Voltammetry

The biosensor developed in this study enabled the evaluation of Hb catalytic activity following exposure to CS by monitoring TMB redox state across varying exposure times. Changes were observed in the oxidation and reduction peaks of TMB, corresponding to peroxide catalysis with iron redox states (Fe^3+^ and Fe^4+^) during oxygen reduction. Normally, the Fe^3+^/Fe^4+^ redox cycle produces well-defined anodic and cathodic peaks in cyclic voltammetry (CV). However, exposure to CS significantly disrupted these signals. A major factor is CO, which binds ferrous Hb (Fe^2+^) with high affinity to form carboxyhemoglobin (HbCO), thereby suppressing or shifting oxidation and reduction peaks. In smokers, CO can occupy up to 38% of Hb compared to only 1–3% in nonsmokers, strongly impairing Hb redox activity [27].

Additional CS components, including nitrogen oxides and cyanide, can further disrupt Hb by binding to the heme iron or destabilizing secondary and tertiary structures. At prolonged exposure times, CV revealed the emergence of double oxidative peaks, likely reflecting conformational changes in Hb subunits and the formation of distinct redox species, such as oxyhemoglobin (Fe^2+^–O_2_), methemoglobin (Fe^3+^), and ferrylhemoglobin (Fe^4+^ = O) [28,29]. These modifications arise from interactions with ROS and reactive nitrogen species (RNS) present in CS. Although partial recovery of Hb redox state was observed after cessation of CS exposure, complete restoration appeared limited, suggesting quasi-irreversible structural alterations [30]. This is consistent with studies reporting reductions in carboxyhemoglobin levels only after >12 h of smoking abstinence, highlighting both reversible and persistent effects of CS on Hb [31,32].

Prolonged exposure of hemoglobin (Hb) to cigarette smoke (CS) leads to the appearance of double oxidative peaks, likely due to conformational changes and the formation of different redox states [33]. While a partial recovery of the Hb redox state can occur after cessation of exposure, some structural damage is irreversible, limiting its ability to return to the reduced state [6]. Reduced carboxyhemoglobin (COHb) levels observed in donors after 12 h of smoking abstinence further support the recovery of Hb function [34].

Exposure to nitric oxide (NO) and nitrites, also components of CS, can further modify Hb redox states. Nitrites, formed from NO and its derivatives in biological fluids, can bind Hb and alter its electrochemical properties, while also reacting with amines such as nicotine to form tobacco-specific nitrosamines (TSNAs), potent carcinogens [35,36]. Our biosensor demonstrated sensitivity to these redox changes, indicating potential for detecting CS-derived compounds. Although current electrochemical biosensors typically focus on nicotine detection or CO monitoring in biological fluids, our approach offers a novel tool for assessing direct Hb redox alterations associated with CS exposure.

### 4.3. Chronoamperometry

Complementary chronoamperometric measurements were conducted to analyze charge-transfer variations under different conditions. A significant decrease in current was observed for Hb treated with sodium azide and for Hb exposed to CS compared to control Hb. Since current intensity directly reflects electron transfer during redox reactions, this decrease indicates inhibition of Hb redox activity by both chemical inhibitors and CS components [37].

Such alterations in electron transfer have also been reported in pathological conditions. For instance, an electrochemical biosensor based on graphene oxide–tellurium nanowires showed decreased currents in thalassemia patients due to β-chain mutations that impair Hb structure and electron transfer [38]. Analogously, CS exposure impairs Hb function at multiple levels: (i) by blocking redox cycling via CO and nitrites that disrupt Fe^2+^/Fe^3+^ transitions, and (ii) by promoting Hb denaturation through ROS overproduction, leading to complete inhibition of peroxidase activity. These findings reinforce the utility of our biosensor in detecting both structural and functional disruptions in Hb induced by CS exposure [39].

In our experiments, we used solutions with a defined pH and optimized analyte concentrations for the CV measurement range. Measurements were performed at room temperature, and repeated tests showed no significant effect on the results. In CV and EIS, the response is mainly governed by diffusion and depends on variables such as analyte concentration, scan rate, and electrode area. Small variations in pH or temperature can alter current values and peak potentials, affecting charge-transfer kinetics and data reproducibility [40,41]. To improve reproducibility, sensitivity, and specificity, we propose developing a microfluidic system integrated with pH and temperature sensors. This setup will allow real-time monitoring and efficient electrode cleaning between samples, ensuring surface stability and consistent electrochemical performance.

In this work, we focused on assessing the time-dependent inhibition of the peroxidase-like activity of methemoglobin (Hb-PLA) after exposure to cigarette smoke. Although, the influence of smoke pressure or concentration on the sensor response was not evaluated in this study. Instead, our aim was to demonstrate the feasibility of using a catalysis based electrochemical biosensor to monitor Hb-PLA as an indirect indicator of its inhibition after smoke exposure. This interpretation was supported by literature describing reactive smoke components that can modify heme proteins and inhibit their catalytic activity [42,43]. Future studies will address this aspect to further characterize the sensor response given the potential of this technique to assess cigarette smoke exposure supporting its applicability in studies related to oxidative stress or environmental biomonitoring.

## 5. Conclusions

The results obtained in this study using the electrochemical biosensor demonstrate that exposure of hemoglobin (Hb) to cigarette smoke (CS) induces a significant inhibition of its peroxidase-like activity (Hb-PLA). This inhibition is time-dependent and quasi-reversible even with prolonged exposure, suggesting partial structural and functional damage to the protein, likely associated with heme group oxidation and the generation of reactive oxygen species (ROS). The biosensor developed here proved to be a sensitive and reliable tool for real-time monitoring of these alterations, positioning it as a promising approach to evaluate the impact of tobacco smoke on biologically relevant proteins such as Hb.

In future studies, this approach could be extended to monitor oxidative damage in biological fluids from active and passive smokers, as well as from individuals exposed to environmental pollutants. It may also be applied to investigate the effects of different types of commercial cigarettes and other tobacco or nicotine products, including cigars, electronic cigarettes, and vaping devices, thus enabling a broader assessment of their oxidative impact.

## Figures and Tables

**Table 1:** Parameters of Hb-PLA peroxidase activity and kinetic characteristics for the analyzed solutions.

Sample	t_CS_ (min)	^+^ %Hb-PLA _máx_	^++^ t_km_ (min)
*Hb_s_	N/A	100	2.0
*Hb_CS1_	1	72	10.1
*Hb_CS5_	5	43	>30
*Hb_CS10_	10	20	>30
*Hb_Az_	N/A	43	17.1

^+^ **%Hb-PLA _máx_** corresponds to the value at t_m_ = 30 min ^++^ t_km_ is the time of Michaelis-Menten’s Hb-PLA activity kinetics.

**Table 2:** Hb-PLA peroxidase activity obtained by spectrophotometry and chronoamperometry measurements for the methemoglobin sample exposed to CS for 10 min.

Method	^+^ %Hb_CS10_-PLA	*r* ^2^
**Spectrophotometry**	13 ± 4.9	0.99
**Biosensor using CA**	25 ± 4.2	0.97

^+^ %Hb_CS10_-PLA corresponds to the value at t_m_ = 600 s.

## Data Availability

Data are contained within the article and Appendix A.

## Abbreviations

The following abbreviations are used in this manuscript:

CS	Cigarette smoke
ROS	Reactive oxygen species
PLA	Peroxidase activity
NaN_3_	Sodium azide
MetHb	Methemoglobin
Hb-PLA	Methemoglobin peroxidase activity
TMB	Tetramethylbenzidine
H_2_O_2_	Hydrogen peroxide
**Hb**_S_	MetHb solutions
**Hb**_CS_	**Hb**_S_ solutions exposed to cigarette smoke
**Hb**_AZ_	**Hb**_S_ solutions with sodium azide
***Hb_S_**	**Hb**_S_ solutions with TMB as positive control
***Hb_CS_**	HbS solutions with TMB exposed to cigarette smoke
***Hb_AZ_**	HbS solutions with sodium azide and TMB as negative control
CV	Cyclic voltammetry
CA	Chronoamperometry
EIS	Electrical impedance spectroscopy

## Appendix A

The table shows the measured data of the redox process kinetics for the positive control (***Hb_s_**), negative control (***Hb_AZ_**), and CS exposure samples **(*Hb_CS1,_ *Hb_CS5,_ *Hb_CS10_**) for the monitoring times.

**Table A1:** Parameters of redox kinetics of Hb-PLA for the analyzed solutions.

	*Hb_s_		*Hb_AZ_		*Hb_CS1_		*Hb_CS5_			*Hb_CS10_		
t_m_ [s]	I_po_	I_pr_	I_po_	I_pr_	I_po_	I_pr_	I_po1_	I_po2_	I_pr_	I_po1_	I_po2_	I_pr_
0	0.193	−20.69	23.96	−78.82	16.41	−102	22.13	-	−147.9	58.37	-	−68.62
30	41.43	−19.15	98.82	−67.75	109.9	−106	64.48	-	−108.4	184.2	-	−39.03
60	29.39	−18.37	124.7	−66.02	174.7	−86.09	164.2	-	−79.63	285.9	90.36	-
90	23.86	−17.43	127.9	−60.74	207.5	−64.21	251.1	-	−36.7	363.1	230	-
120	20.92	−16.67	133.9	−55.55	219.2	−51.83	310.5	230.6	−10.14	449.9	417	-
180	17.91	−16.05	135	−56.69	232.3	−47.1	352	367	-	569.9	572.1	-
300	13.25	−16.6	135.5	−57.34	181.2	−68.74	280.1	257.4	−20.68	652.5	671.5	-
600	4.73	−19.15	81.21	−78.26	33.97	−90.57	91.84	-	−93.31	793	888.3	-
900	3.55	−19.81	45.53	−88.22	18.42	−82.08	48.4	-	−99.11	233.8	-	−68.21
1200	2.82	−19.93	42.3	−86.89	28.95	−75.95	42.04	-	−97.32	74.17	-	−86.58
1800	1.63	−19.87	30.71	−85.9	53.9	−71.82	25.89	-	−92.27	30.48	-	−84.39

## References

[1] Alayash A.I. (1999). Hemoglobin-based blood substitutes: Oxygen carriers, pressor agents, or oxidants?. Nat. Biotechnol..

[2] Ahmed M.H., Ghatge M.S., Safo M.K. (2020). Hemoglobin: Structure, Function and Allostery. Subcell. Biochem..

[3] Eaton W.A. (2022). Impact of hemoglobin biophysical studies on molecular pathogenesis and drug therapy for sickle cell disease. Mol. Asp. Med..

[4] Reeder B.J. (2010). The redox activity of hemoglobins: From physiologic functions to pathologic mechanisms. Antioxid. Redox Signal..

[5] Caliri A.W., Tommasi S., Besaratinia A. (2021). Relationships among smoking, oxidative stress, inflammation, macromolecular damage, and cancer. Mutat. Res. Rev. Mutat. Res..

[6] Seo Y.S., Park J.M., Kim J.H., Lee M.Y. (2023). Cigarette Smoke-Induced Reactive Oxygen Species Formation: A Concise Review. Antioxidants.

[7] Alayash A.I. (2019). Mechanisms of Toxicity and Modulation of Hemoglobin-Based Oxygen Carriers. Shock.

[8] Pryor W.A., Stone K. (1993). Oxidants in cigarette smoke. Radicals, hydrogen peroxide, peroxynitrate, and peroxynitrite. Ann. N. Y. Acad. Sci..

[9] Munteanu I.G., Apetrei C. (2022). A Review on Electrochemical Sensors and Biosensors Used in Assessing Antioxidant Activity. Antioxidants.

[10] Zhang L., Jiang X., Wang E., Dong S. (2005). Attachment of gold nanoparticles to glassy carbon electrode and its application for the direct electrochemistry and electrocatalytic behavior of hemoglobin. Biosens. Bioelectron..

[11] Elewi A.S., Al-Shammaree S.A.W., Al Sammarraie A.K.M.A. (2020). Hydrogen peroxide biosensor based on hemoglobin-modified gold nanoparticles–screen printed carbon electrode. Sens. Bio-Sens. Res..

[12] Abd-Elsabour M., Alsoghier H.M., Alhamzani A.G., Abou-Krisha M.M., Yousef T.A., Assaf H.F. (2022). A Novel Electrochemical Sensor for Detection of Nicotine in Tobacco Products Based on Graphene Oxide Nanosheets Conjugated with (1,2-Naphthoquinone-4-Sulphonic Acid) Modified Glassy Carbon Electrode. Nanomaterials.

[13] Parate K., Karunakaran C., Claussen J.C. (2019). Electrochemical cotinine sensing with a molecularly imprinted polymer on a graphene-platinum nanoparticle modified carbon electrode towards cigarette smoke exposure monitoring. Sens. Actuators B Chem..

[14] Mirani A., Kianfar E., Maleknia L., Javanbakht M. (2024). Recent advances in nicotine electrochemical biosensors: A review. Case Stud. Chem. Environ. Eng..

[15] Biswas P., Seal P., Sikdar J., Haldar R. (2021). Oxidative degradation perturbs physico-chemical properties of hemoglobin in cigarette smokers: A threat to different biomolecules. Inhal. Toxicol..

[16] Alayash A.I., Wilson M.T. (2022). Hemoglobin can Act as a (Pseudo)-Peroxidase in vivo. What is the Evidence?. Front. Mol. Biosci..

[17] Wilson M.T., Reeder B.J. (2022). The peroxidatic activities of Myoglobin and Hemoglobin, their pathological consequences and possible medical interventions. Mol. Asp. Med..

[18] Hua X., Yang Z., Wang Z., Xie X., Zhou Z., Yang X., Deng K., Huang H. (2020). Rapid modification of hemoglobin heme to form enhanced peroxidase-like activity for colorimetric assay. Biosens. Bioelectron. X.

[19] Piantadosi C.A. (2008). Carbon monoxide, reactive oxygen signaling, and oxidative stress. Free Radic. Biol. Med..

[20] Reznick A.Z., Klein I., Eiserich J.P., Cross C.E., Nagler R.M. (2003). Inhibition of oral peroxidase activity by cigarette smoke: In vivo and in vitro studies. Free Radic. Biol. Med..

[21] Liu G., Amin S., Okuhama N.N., Liao G., Mingle L.A. (2006). A quantitative evaluation of peroxidase inhibitors for tyramide signal amplification mediated cytochemistry and histochemistry. Histochem. Cell Biol..

[22] Shaw M., Mitchell R., Dorling D. (2000). Time for a smoke? One cigarette reduces your life by 11 minutes. BMJ.

[23] Schimmel J., George N., Schwarz J., Yousif S., Suner S., Hack J.B. (2018). Carboxyhemoglobin Levels Induced by Cigarette Smoking Outdoors in Smokers. J. Med. Toxicol..

[24] Furtmüller P.G., Zederbauer M., Jantschko W., Helm J., Bogner M., Jakopitsch C., Obinger C. (2006). Active site structure and catalytic mechanisms of human peroxidases. Arch. Biochem. Biophys..

[25] Malenica M., Prnjavorac B., Bego T., Dujic T., Semiz S., Skrbo S., Gusic A., Hadzic A., Causevic A. (2017). Effect of Cigarette Smoking on Haematological Parameters in Healthy Population. Med. Arch..

[26] Yunus G., Singh R., Raveendran S., Kuddus M. (2023). Electrochemical biosensors in healthcare services: Bibliometric analysis and recent developments. PeerJ.

[27] Rifkind J.M., Nagababu E., Ramasamy S., Ravi L.B. (2003). Hemoglobin redox reactions and oxidative stress. Redox Rep..

[28] Tang J., Tang D., Su B., Li Q., Qiu B., Chen G. (2011). Nanosilver-penetrated polyion graphene complex membrane for mediator-free amperometric immunoassay of alpha-fetoprotein using nanosilver-coated silica nanoparticles. Electrochim. Acta.

[29] Jara-Palacios M.J., Begines E., Heredia F.J., Escudero-Gilete M.L., Hernanz D. (2024). Effectiveness of Cyclic Voltammetry in Evaluation of the Synergistic Effect of Phenolic and Amino Acids Compounds on Antioxidant Activity: Optimization of Electrochemical Parameters. Foods.

[30] Dorey A., Scheerlinck P., Nguyen H., Albertson T. (2020). Acute and Chronic Carbon Monoxide Toxicity from Tobacco Smoking. Mil. Med..

[31] Doyle M.P., Hoekstra J.W. (1981). Oxidation of nitrogen oxides by bound dioxygen in hemoproteins. J. Inorg. Biochem..

[32] Way J.L. (1984). Cyanide intoxication and its mechanism of antagonism. Annu. Rev. Pharmacol. Toxicol..

[33] Ghosh A., Banerjee S., Mitra A., Muralidharan M., Roy B., Banerjee R., Mandal A.K., Chatterjee I.B. (2016). Interaction of p-benzoquinone with hemoglobin in smoker’s blood causes alteration of structure and loss of oxygen binding capacity. Toxicol. Rep..

[34] Boehm R.E., Arbo B.D., Leal D., Hansen A.W., Pulcinelli R.R., Thiesen F.V., Balsan A.M., Onsten T.G.H., Gomez R. (2018). Smoking fewer than 20 cigarettes per day and remaining abstinent for more than 12 hours reduces carboxyhemoglobin levels in packed red blood cells for transfusion. PLoS ONE.

[35] Fini H., Kerman K. (2020). Revisiting the nitrite reductase activity of hemoglobin with differential pulse voltammetry. Anal. Chim. Acta.

[36] Mocniak L.E., Bitzer Z.T., Trushin N., Richie J.P. (2022). Effects of tobacco nitrate content on free radical levels in mainstream smoke. Free Radic. Biol. Med..

[37] Chen H., Cui L., Jiang X.Y., Pang Y.Q., Tang G.L., Hou H.W., Jiang J.H., Hu Q.Y. (2012). Evaluation of the cytotoxicity of cigarette smoke condensate by a cellular impedance biosensor. Food Chem. Toxicol..

[38] Sana Rafiq H., Fatima B., Hussain D., Mohyuddin A., Majeed S., Manzoor S., Imran M., Nawaz R., Shabbir S., Mukhtar S. (2021). Selective electrochemical sensing of hemoglobin from blood of β-thalassemia major patients by tellurium nanowires-graphene oxide modified electrode. Chem. Eng. J..

[39] Duong C., Seow H.J., Bozinovski S., Crack P.J., Anderson G.P., Vlahos R. (2010). Glutathione peroxidase-1 protects against cigarette smoke-induced lung inflammation in mice. Am. J. Physiol. Lung Cell Mol. Physiol..

[40] Arslan H., Özdemir M., Zengin H., Zengin G. (2012). Glucose Biosensing at Carbon Paste Electrodes Containing Polyaniline-Silicon dioxide Composite. Int. J. Electrochem. Sci..

[41] Schinagl M., Fasching M., Höschele P., Ellersdorfer C. (2025). Impact of temperature on Li-ion battery impedance and compensation strategies. J. Power Sources.

[42] Mitra A., Mandal A.K. (2018). Conjugation of para-benzoquinone of Cigarette Smoke with Human Hemoglobin Leads to Unstable Tetramer and Reduced Cooperative Oxygen Binding. J. Am. Soc. Mass Spectrom..

[43] Méndez-Alvarez E., Soto-Otero R., Sánchez-Sellero I., López-Rivadulla Lamas M. (1998). In vitro inhibition of catalase activity by cigarette smoke: Relevance for oxidative stress. J. Appl. Toxicol..

